# Line Immunoblot Assay for Tick-Borne Relapsing Fever and Findings in Patient Sera from Australia, Ukraine and the USA

**DOI:** 10.3390/healthcare7040121

**Published:** 2019-10-21

**Authors:** Jyotsna S. Shah, Song Liu, Iris Du Cruz, Akhila Poruri, Rajan Maynard, Mariia Shkilna, Mykhaylo Korda, Ivan Klishch, Stepan Zaporozhan, Kateryna Shtokailo, Mykhaylo Andreychyn, Raphael B. Stricker, Ranjan Ramasamy

**Affiliations:** 1 IGeneX Inc., 556 Gibraltar Drive, Milpitas, CA 95035, USA; icruz@igenex.com (I.D.C.); aporuri@igenex.com (A.P.); 2 ID-FISH Technology Inc., 556 Gibraltar Drive, Milpitas, CA 95035, USA; steells9508@gmail.com (S.L.); rramasamy@idfishtechnology.com (R.R.); 3 Stanford Genome Technology Center, 3165 Porter Drive, Palo Alto, CA 94304, USA; maynard2@stanford.edu; 4 I. Horbachevsky Ternopil National Medical University, Voli Square, 1, 46002 Ternopil, Ternopil‘s’ka oblast, Ukraine; nadiya20743@gmail.com (M.S.); korda@tdmu.edu.ua (M.K.); klishch@tdmu.edu.ua (I.K.); zaporozhan@tdmu.edu.ua (S.Z.); yavorska.kb@gmail.com (K.S.); andreychyn@tdmu.edu.ua (M.A.); 5 Union Square Medical Associates, 450 Sutter Street, Suite 1504, San Francisco, CA 94108, USA; rstricker@usmamed.com

**Keywords:** borreliosis, line immunoblots, Lyme disease, relapsing fever, relapsing fever *Borreliae*, serological diagnosis

## Abstract

Tick-borne relapsing fever (TBRF) is caused by spirochete bacteria of the genus *Borrelia* termed relapsing fever *Borreliae* (RFB). TBRF shares symptoms with Lyme disease (LD) caused by related Lyme disease *Borreliae* (LDB). TBRF and LD are transmitted by ticks and occur in overlapping localities worldwide. Serological detection of antibodies used for laboratory confirmation of LD is not established for TBRF. A line immunoblot assay using recombinant proteins from different RFB species, termed TBRF IB, was developed and its diagnostic utility investigated. The TBRF IBs were able to differentiate between antibodies to RFB and LDB and had estimated sensitivity, specificity, and positive and negative predictive values of 70.5%, 99.5%, 97.3%, and 93.4%, respectively, based on results with reference sera from patients known to be positive and negative for TBRF. The use of TBRF IBs and analogous immunoblots for LD to test sera of patients from Australia, Ukraine, and the USA with LD symptoms revealed infection with TBRF alone, LD alone, and both TBRF and LD. Diagnosis by clinical criteria alone can, therefore, underestimate the incidence of TBRF. TBRF IBs will be useful for laboratory confirmation of TBRF and understanding its epidemiology worldwide.

## 1. Introduction

Relapsing fever (RF) and Lyme disease (LD) in humans are caused by two related groups of spirochete bacteria of the genus *Borrelia* known as relapsing fever *Borreliae* (RFB) and Lyme disease *Borreliae* (LDB), respectively. Both groups of *Borreliae* are transmitted by arthropod vectors. New species continue to be discovered and added to the greater than 20 species of *Borreliae* in each group [1,2]. LD is considered to be the most prevalent tick-borne disease in North America and Western Europe, and the disease has been reported on every continent except Antarctica [1,2,3]. LD in the USA is caused mainly by *Borrelia burgdorferi* sensu stricto (Bbss) [4,5]. *Borrelia bissettii* [6] and *B. mayonii* [7] also cause LD in the USA, while *B. afzelii*, *B. garinii*, and Bbss are largely responsible for LD in Europe and Asia [8]. Hard ticks of the *Ixodes* species are the principal vectors responsible for transmitting LDB from animal reservoirs to humans in the USA, Europe, and Asia [3]. The transmission of other tick-borne diseases and persons with LD-like symptoms have been observed in Australia but local transmission of LDB or RFB to humans is controversial [9,10,11].

Relapsing fever, which was historically widespread in the world [12], is initiated by the transmission of RFB from animal reservoirs by ticks causing tick-borne relapsing fever or TBRF and human to human transmission by the human body louse causing louse-borne relapsing fever or LBRF. There is now increasing recognition that TBRF has been relatively neglected as a disease in North America, Europe and elsewhere [13,14,15,16]. RFB species that are best known to cause TBRF in the USA are *B. hermsii, B. miyamotoi, B. parkeri*, and *B. turicatae* [13]. However, other RFB species that cause TBRF continue to be identified, for example, a patient infected with *B. johnsonii*-like species, previously found only in bat ticks, was recently identified in Wisconsin [17]. TBRF has also been reported in Central and South America [18]. *Borrelia hispanica* [19], *B. persica* [20], and *B. miyamotoi* [21] are important causes of TBRF in Europe and Asia, and *B. hispanica*, *B. crocidurae*, and *B. duttonii* are important causes of TBRF in Africa [22]. While most RFB are transmitted by soft ticks of the genus *Ornithodoros, B. miyamotoi* can be transmitted by the same hard ticks of the genus *Ixodes* that transmit LDB [13,23]. Another RFB species, *Borrelia lonestari*, is reported to be transmitted by a different hard tick, *Amblyomma americanum*, in southern USA [24]. LD and TBRF occur in overlapping localities where their vectors are present [13,23,25]. 

LBRF, caused by *B. recurrentis*, and transmitted by the human body louse *Pediculus humanus humanus*, is not endemic in the USA and Western Europe [12,15], however, LBRF remains prevalent in resource-constrained countries of North East Africa [22] and has recently been observed among migrants and refugees arriving in Europe from these countries [26]. 

LD and TBRF share several nonspecific clinical signs and symptoms, for example, headache, myalgia, arthralgia, fever, and chills. This makes differential clinical diagnosis a challenge to physicians [27]. LD and TBRF differ clinically in that relapses of fever are more common in TBRF [28] while an erythema migrans rash associated with an infected tick bite is more common in LD [29]. Recurrent blood spirochetemia and consequently febrile episodes in TBRF are believed to be caused by the bacteria sequentially expressing members of two families of outer membrane proteins with varying antigenicity that serve to subvert host-protective antibody responses [30,31]. Advanced LD and TBRF can cause different pathologies [13,28,29,32]. The correct diagnosis of LD and TBRF is, therefore, relevant for management of patients and for advancing knowledge about the epidemiology of the two tick-borne diseases. 

Laboratory diagnosis of TBRF by microscopic examination of blood smears and PCR is confounded by bacteria leaving the bloodstream to sequester in tissues. Acute fever episodes in TBRF are characterized by high blood spirochetemia that can reach greater than 10^5^ *Borrelia* per ml of blood [13,28]. PCR and microscopy are expected to be most effective for diagnosis during this period, however, the examination of Giemsa stained blood smears has been reported to be insensitive as compared with PCR for *B. miyamotoi* infections [33]. Culturing bacteria for diagnosing TBRF is difficult even when there is high blood spirochetemia because *Borreliae* do not readily adapt to culture. Detection of antibodies to specific LDB antigens is presently the most important laboratory diagnostic method for LD. A two-tier enzyme-linked immunosorbent assay (EIA) followed by an IgG or IgM Western blot (WB) assay on whole Bbss cell lysates is presently recommended by the US Centers for Disease Control and Prevention (CDC) for the serological diagnosis of Bbss [29]. The algorithms recommended by the CDC for identifying positive reactions in IgG and IgM WBs are based on ten specific Bbss proteins [29]. A form of WB that uses purified recombinant proteins, termed line immunoblot or IB, which includes all ten Bbss proteins utilized in the CDC WB diagnostic algorithms, as well as additional proteins from other LDB species, has been developed for serological detection of IgG and IgM antibodies in LD [34]. The IB offers many advantages over WB as a laboratory test to assist the diagnosis of LD and, as a one-step test, the LD IB showed comparable or better clinical diagnostic results than the two-tier test for LD [34]. 

Antigenic cross-reactivity between RFB and LDB has been observed in serological tests employing whole cell lysates as antigens [1,35,36,37], because of their close phylogenetic relationship. However, a glycerophosphodiester diesterase (GlpQ) periplasmic enzyme present in RFB [38], including *B. recurrentis* [39], but not reportedly in LDB, has been used in EIAs and WBs for diagnostic purposes. Another putative RFB-specific candidate for differential diagnosis is a surface exposed lipoprotein termed the *Borrelia* immunogenic protein A or BipA [40,41]. A third potential RFB-specific candidate antigen is another surface lipoprotein that binds factor H and a related factor H-like protein (termed the factor H binding protein or fHbp) and helps protect RFB from human complement-mediated killing through activation of the alternative pathway of complement [42,43,44]. Additionally, GlpQ and proteins of approximate molecular masses similar to BipA (70-75kDa) and fHbp (20-23kDa) were identified as being able to detect RFB-specific antibodies in WBs performed on whole cell lysates of *B. hermsii* and *B. turcica* [25,45]. 

Analogous to the use of purified recombinant proteins as antigens in line IBs for the serological diagnosis of LD [34], we have developed, for the first time, line IBs based on purified recombinant RFB proteins for the serological diagnosis of TBRF termed TBRF IBs. Sera of patients with LD-like symptoms from Australia, Ukraine, and the USA were subsequently tested with the TBRF IBs. 

## 2. Materials and Methods 

### 2.1. Human Sera for Assessing Clinical Specificity of TBRF Immunoblots

A total of 212 human sera expected to be negative for TBRF were obtained from the CDC, College of American Pathologists, New York State Department of Health, New York Biologics (Southampton, NY, USA), Columbia University (New York, NY, USA) and IGeneX (Table 1). The IGeneX test samples were leftover sera received for routine testing for tick-borne diseases that would otherwise be discarded. 

### 2.2. Rabbit Antisera for Testing Antigenic Cross-Reactivity between RFB and LDB Proteins

Individual rabbit antisera were raised by multiple injections of whole cell lysates against the LDB species *B. afzelii*, Bbss strain B31, Bbss strain 297, *B. californiensis*, *B. garinii*, *B*. *spielmanii*, and *B. valaisiana* grown in culture, as previously described [34]. The seven sera recognized all the recombinant antigens used for scoring positive reactions in LD IBs [34]. Similarly, individual rabbit antisera were produced against whole cell lysates of the culture-grown RFB species *B. coriaceae*, *B. hermsii*, and *B. turcica*. The antisera to *B. coriaceae* and *B. hermsii* when tested previously weakly recognized p41 flagellin B (FlaB) from LDB, but did not react with all other antigens used in LD IBs [34].

### 2.3. Human Sera for Assessing Clinical Sensitivity of TBRF Immunoblots

A set of fifty one left over sera received for routine tick-borne disease testing at IGeneX that were confirmed positive for RFB by qPCR and would otherwise be discarded, were anonymized and tested by TBRF IBs. These were different from the reference IGeneX sera described in Table 1. Subsequent serum samples for testing were received from 16 of these patients, after an interval of 5 to 53 weeks and were similarly tested on TBRF IBs. 

### 2.4. Sera from Patients with LD-Like Symptoms

The following serum samples from patients with LD-like symptoms from Australia, Ukraine, and the USA were tested by LD and TBRF IBs:

#### 2.4.1. Australia

One hundred left over and decoded serum samples that would otherwise be discarded, received from Australia in the period September 2015 to December 2017 for routine testing for tick-borne diseases at IGeneX, were tested by TBRF and LD IBs.

#### 2.4.2. Ukraine

One hundred and twenty-one anonymized serum samples from patients residing in Western Ukraine, were tested as part of the Ukrainian–Polish research project, “Investigation of Epidemiology, Pathogenesis, Clinics, and Prophylaxis of Borreliosis”. The research was approved by the Bioethics Committee of I. Ya. Horbachevsky Ternopil National Medical University (No. 30 dated 01/09/2015). Sera were received at IGeneX in October 2016 and tested on TBRF and LD IBs. 

#### 2.4.3. USA

A total of 1158 sera were received at IGeneX in the period April to July 2018 for tick-borne diseases testing, including TBRF and LD IBs. The results were anonymized for analysis.

### 2.5. Storage, Testing Approach, and Ethical Considerations with Human Sera

Sera were stored at 4 °C up to one week and at –20 °C for longer storage. Tests on human sera for investigating sensitivity and specificity of TBRF immunoblots were performed by laboratory personnel without prior knowledge of the expected results in the same manner as clinical samples from patients with suspected LD [34]. 

Institutional review board approval and consent from patients for samples received at IGeneX were not required because the results presented are based on retrospective analysis of de-identified clinical samples routinely received at IGeneX for testing for tick-borne diseases and the use of leftover de-identified sera that would otherwise have been discarded. The Ukraine sera used in this study were collected for research use with the approval of the Institutional Bioethics Committee (see Section 2.4.2).

### 2.6. PCR Detection of RFB in Blood 

DNA was purified with the Generation Capture Column kit (Qiagen, Valencia, CA, USA) from patient blood as previously described [47] and then subjected to quantitative PCR (qPCR). A fragment of 16S rDNA was amplified with *Borrelia* genus specific primers (forward primer: 5’- GGC GTA AAG GGT GAG TAG G -3’ and reverse primer: 5’- CAG ATT CCA CCC TTA CAC CAG -3’) and then quantitated with a dye-labelled RFB-specific probe (5’- TCC CCT ATC AGA CTC TAG TCA TGC AGT TTC -3’). The qPCR detected all three RFB species tested (*B. coriaceae*, *B. hermsii*, *B. miyamotoi*) and was predicted by BLASTn analysis to react with all RFB species whose 16S rRNA sequences are in the National Centre for Biotechnology Information (NCBI, Bethesda, MD, USA) database. It did not detect Bbss B31 or five other LDB species tested (*B. afzelii*, *B. californiensis*, *B. garinii*, *B. spielmanii*, *B. valaisiana*). The qPCR also did not detect several other common human blood-borne bacterial and protozoan pathogens. The limit of detection was estimated to be 69 *B. hermsii* per 200 μl of blood used for the qPCR test. 

### 2.7. Preparation of Antigen Strips for IBs

All recombinant antigens for IBs were prepared by cloning hybrid gene constructs or portions of the selected genes into pET vectors, expressing the proteins in *Escherichia coli* (GenScript, Piscataway, NJ, USA), and isolating them to >90% purity, as described previously [34]. 

#### 2.7.1. Antigens for LD Immunoblots

The LD IBs included all ten Bbss proteins used in scoring WBs by the established CDC criteria [29] together with the additional antigens OspA, OspB, VlsE, and C6, as previously described [34]. Because sensitivity is improved by using multiple Bbss isolates in WBs [48,49], proteins from two Bbss strains, as well as other US and European LDB, were used for scoring reactions in LD IBs [34]. 

#### 2.7.2. Antigens in TBRF Immunoblots

Recombinant proteins from different RFB species were included for the detection of each of the four target antigens, BipA, GlpQ, fHbp, and FlaB in TBRF IBs [50], in an analogous manner to the approach taken for LD IBs by [34]. This was done in order to improve the sensitivity of detection for different RFB species, including *B hermsii, B. miyamotoi*, and *B. turicatae*. Five recombinant proteins for BipA, four for GlpQ, two for fHbp, and three for FlaB were, therefore, used in the TBRF IBs.

Two additional proteins tested as possible antigens in the TBRF IBs were *B. hermsii* BmpA (one recombinant protein) and *B. hermsii* OspC (one recombinant protein). The antigen strips for TBRF IBs were prepared with control and test antigens, essentially as previously described for LD IBs [34]. 

### 2.8. Detection of Antibodies in Patient Sera with TBRF and LD Immunoblots 

Both IgG and IgM antibodies were detected in LD and TBRF IBs using the previously described procedure for LD IBs [34]. 

#### 2.8.1. Controls for Immunoblots 

Alkaline phosphatase-conjugated rabbit antibody to the 39/93 kDa LDB antigens (Strategic Biosciences, Stow, MA, USA) diluted in human serum was used as a calibration control in both TBRF and LD IBs and bands with lower intensity than the calibration control were considered to be negative [34]. The two proteins used as controls in both IBs were a mixture of human IgM and IgG (Sigma, St. Louis, MO, USA) for detecting the addition of alkaline phosphatase conjugated anti-human antibodies (C1), and Protein L (Sigma, St. Louis, MO, USA) for detecting the addition of human serum (C2), as previously described [34]. 

Immune rabbit sera for use as a marker control in TBRF IBs were prepared by immunizing individual rabbits separately with each of the recombinant antigens from the different RFB species used in TBRF IBs (Pacific Immunology, Ramona, CA, USA). The rabbit sera were then pooled and used at a dilution of 10^−4^ in a single control IB strip. Alkaline phosphatase-labelled goat anti-rabbit whole IgG (KPL, Gaithersburg, MD, USA) was used as the secondary antibody. 

#### 2.8.2. Scoring of Reactivity of Patient Sera in LD and TBRF Immunoblots

The reactions in LD IBs were scored according to the established CDC criteria for detecting specific antigens in IgG and IgM Western blots [29] but allowing for recognition of proteins derived from different LDB species in the IBs to improve sensitivity of detection as previously described [34]. 

IgG and IgM antibodies were similarly detected in TBRF IgG and IgM IBs. The scoring algorithm for TBRF antibodies was developed as described below in Section 3.2, after analyzing the sensitivity and specificity of reactions with panels of TBRF positive and TBRF negative human sera described in Section 2.1 and Section 2.3.

### 2.9. TBRF Immunoblots with Rabbit Antisera for Investigating Antigenic Cross-Reactivity between LDB and RFB Proteins

The ten rabbit antisera against LDB and RFB described in Section 2.2 were used in TBRF IBs to investigate cross-reactivity between LDB and RFB antigens essentially as previously described for LD IBs [34]. Antigen strips for TBRF IBs were prepared and reacted with the different rabbit antisera followed by alkaline phosphatase-labelled goat anti-rabbit whole IgG (KPL, Gaithersburg, MD, USA) as the secondary antibody. Additional strips were reacted with negative control human serum as previously described for human serum in LD IBs [34] and the pooled rabbit antiserum to all the RFB test antigens described in Section 2.8.1 as a positive marker control. 

### 2.10. Statistical Analysis 

The diagnostic sensitivity, specificity, positive predictive value (PPV), negative predictive value (NPV), and their 95% confidence intervals were determined with an online statistical calculator [51].

### 2.11. Protein Sequence Homology Analysis

Sequence homologies were determined by the NCBI online BLASTp analysis [52].

## 3. Results

### 3.1. Antigenic Cross-Reactivity between the LDB and RFB proteins

**Figure 1:**
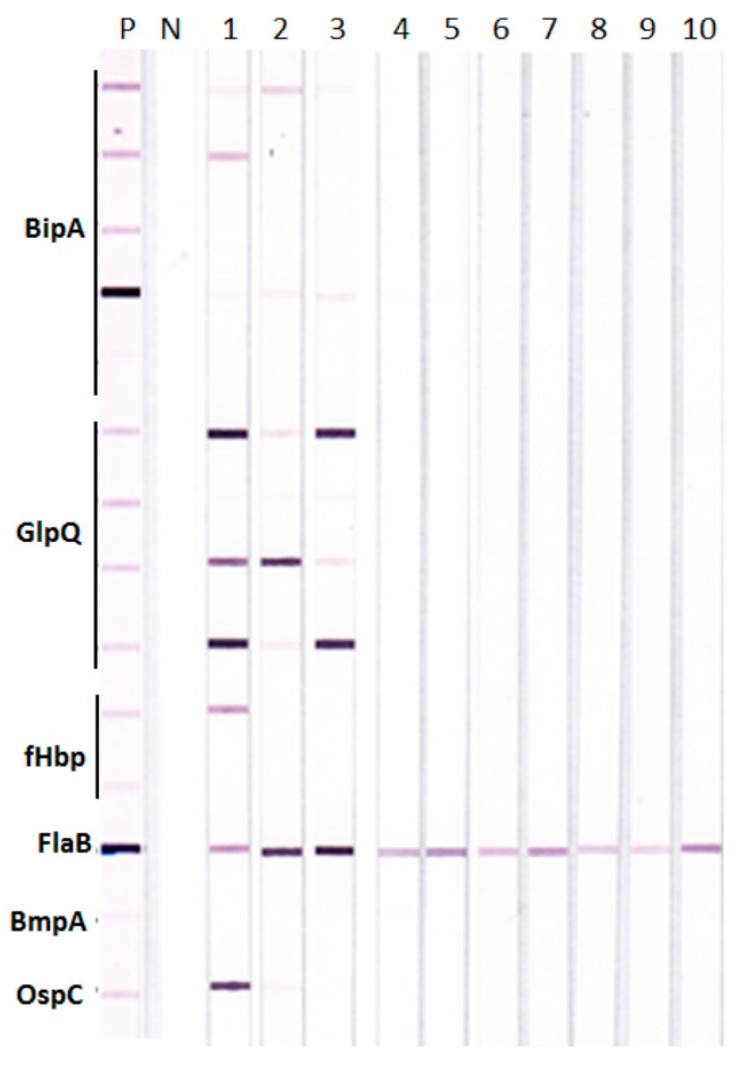
Tick-borne relapsing fever immunoblots (TBRF IBs) with rabbit antisera to different *Borrelia* species. Rabbit antisera produced against different relapsing fever *Borreliae* (RFB) (lanes 1–3) and Lyme disease *Borreliae* (LDB) (lanes 4–10) were tested individually on TBRF IBs. Rabbit antibodies to: Lane 1, *B. hermsii*; lane 2, *B. turcica*; lane 3, *B. coriaceae*; lane 4, Bbss strain B31; lane 5, Bbss strain 297; lane 6, *B. afzelii*; lane 7, *B. garinii*; lane 8, *B. californiensis*; lane 9, *B. spielmanii*; and lane 10, *B. valaisiana*. P, positive control pooled rabbit antiserum and N, negative control human serum.

Possible antigenic cross-reactivity between LDB and RFB proteins that could give false positive reactions in TBRF IBs was investigated with rabbit antisera raised against whole cell lysates of different LDB and RFB species. The results show that antisera against different LDB species, including two different Bbss strains B31 and 297, reacted only with the FlaB derived from RFB and none of the other test RFB antigens in TBRF IBs (Figure 1). Antigenic cross-reactivity between LDB and RFB FlaB proteins is consistent with high sequence homology of >90% between the two proteins shown in the BLASTp analysis (Supplementary Table S1).

The rabbit antisera against the three RFB species also demonstrated variable cross-reactions among BipA, GlpQ, and fHbp proteins from different RFB species (Figure 1). For example, the strongest reactions were seen with the rabbit antiserum against *B. hermsii* to two BipA, three GlpQ, and one fHbp proteins (Figure 1, lane 1); with the rabbit antiserum against *B. turcica* to one BipA and two GlpQ proteins (Figure 1, lane 2); and with the rabbit antiserum against *B. coriaceae* to one BipA and three GlpQ proteins (Figure 1, lane 3). This variable cross-reactivity is consistent with the varying degrees of sequence homology within BipA, GlpQ, and fHbp proteins from different RFB species as shown by BLASTp analysis in comparison to *B. hermsii* proteins (Supplementary Table S1). Analysis of BipA, GlpQ, fHbp, and FlaB sequences further suggests that (i) sequence homologies between *E. coli*, the *Treponema* spirochetes and RFB FlaB were <40%, whereas that between RFB and LDB FlaB was >90%; (ii) BipA has moderate homology within RFB, <30% homology with LDB, and no significant homology with *Treponema* and *E. coli;* (iii) GlpQ shows high homology (85%–90%) within RFB, weaker homology with *Treponema* and *E. coli* (<50%), and insignificant homology with LDB; and (iii) fHbp has moderate homology within RFB, no significant homology with Bbss, *Treponema*, and *E. coli* but shows weak homology with European LDB species other than Bbss. 

### 3.2. Scoring Algorithm to Optimize Specificity and Sensitivity of the TBRF IB Assay

#### 3.2.1. Specificity of Detecting Antibodies in TBRF IBs

The 212 reference human sera expected to be negative for antibodies to TBRF (Table 1) were tested by TBRF IgG and IgM IBs and their reactivity with the different combinations of antigens in the two IBs was analyzed for optimizing the specificity of detection of antibodies. 

The detection of either IgG or IgM antibodies against FlaB, as well as any two out of the three antigens BipA, GlpQ, and fHbp, gave the best specificity for detecting antibodies to RFB in the IB assays. Detection of either IgG or IgM antibodies to one or more proteins within each antigen type was regarded as a positive reaction for that antigen type. Using this algorithm a single serum, out of the 212 expected to be negative, reacted positively in the TBRF IB assay. This was a serum with an elevated IgG concentration in the autoimmunity set of reference sera.

#### 3.2.2. Sensitivity of Detecting Antibodies in TBRF IBs

**Figure 2:**
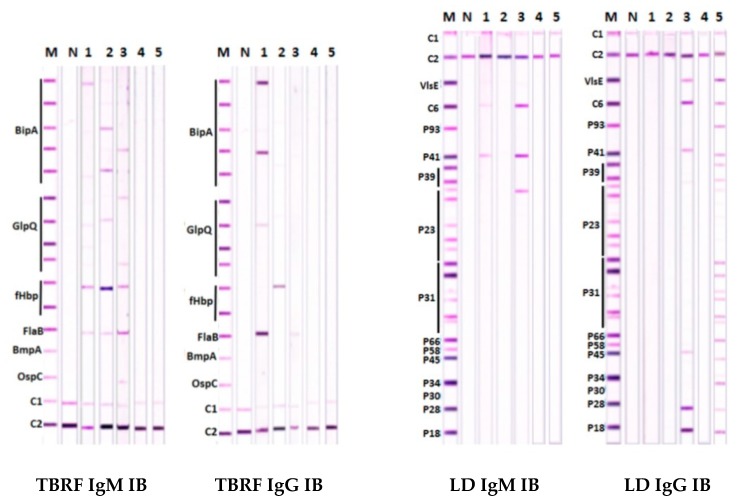
TBRF and LD IgM and IgG immunoblots with representative patient sera. Results obtained with sera from five patients in IgM and IgG IBs for TBRF and LD, where antibody reactivity was scored as positive according to criteria described in Section 2.8.2 for LD IBs and Section 3.2.1 for TBRF IBs. M: positive control (rabbit antiserum for TBRF IBs and human serum for LD IBs); N: negative control (human sera for TBRF and LD IBs); C1: conjugate control; and C2: serum control. The positions of the different target antigens used in the IB strips are indicated.

Sera that were positive by qPCR for RFB were used to establish the sensitivity of detecting antibodies in TBRF IBs. The results of testing sera from 51 patients that were positive for RFB by qPCR are shown in Table 2. Examples of positive and negative TBRF IBs with patient sera are shown in Figure 2 in Section 3.3.

The algorithm, described in Section 3.2.1, for detecting antibodies against FlaB plus any two out of the three antigens BipA, GlpQ, and fHbp, can be applied individually to IgM and IgG TBRF IBs. However, applying the same criterion for the detection of either IgM or IgG antibodies to any of the four antigens led to a better sensitivity of detection. Thus four RFB-PCR positive patients were missed by not considering the detection of either IgG or IgM antibodies against the four scoring antigens using the algorithm described in Section 3.2.1.

The results also show that three of the patients who tested negative in the TBRF IBs for either IgG or IgM antibodies with the first serum became positive when a second serum sample was tested, so that 39 patients out of 51 now became positive in TBRF IBs (comparison A and B in Table 2). This was due to the detection of IgM antibodies in the three second sera which were provided six, nine, and 18 weeks, respectively, after the first sera that were negative in TBRF IBs. Improved IgG responses were, however, observed in the second serum as compared with the first serum in 10 of the 16 samples. 

#### 3.2.3. Clinical Parameters of Antibody Detection in TBRF Immunoblots

The estimated clinical parameters determined with the reference human sera described in Section 2.1 and Section 2.3 using the selected scoring algorithm adopted in Section 3.2.1 are listed in Table 3. The clinical parameters were improved when the second serum sample was used in place of the first serum sample in 16 of the 51 patients with TBRF confirmed by PCR. 

### 3.3. Findings with TBRF and LD IB Assays in Patients with LD-Like Symptoms

TBRF and LD IB results with sera from Australia, Ukraine, and the USA in patients with LD-like symptoms using the scoring algorithms described in Section 2.8.2 and Section 3.2.1 are shown in Table 4. Reactions in (i) TBRF IBs only, (ii) LD IBs only, and (iii) both TBRF and LD IBs were observed in all three countries.

TBRF IBs showing representative reactions with patient sera in each of the categories (i) to (iii) above are presented in Figure 2.

Sera of both patients in lanes 1 and 2 were only positive for antibodies to RFB. Serum in lane 3 was positive for antibodies to both RFB and LDB. Serum in lane 4 was negative for antibodies to both RFB and LDB. Serum in lane 5 was only positive for antibodies to LDB. Serum tested in lane 1 showed a stronger reaction with IgG than IgM antibodies, whereas sera in lanes 2 and 3 had better reaction with IgM than IgG antibodies in TBRF IBs, demonstrating the importance of assaying both antibody classes in patients. None of the three TBRF IB positive sera recognized BmpA, while only serum in lane 3, which reacted positively in both TBRF and LD IBs, recognized OspC. The TBRF IB positive sera in lanes 1 and 2 showed no recognition of any of the scoring LDB proteins in LD IBs except for FlaB, a result consistent with previous findings with rabbit antisera against RFB species [34]. 

Conversely, the patient serum with antibodies to LDB in lane 5 did not react with any of the scoring RFB-specific antigens in TBRF IBs, including FlaB. The results with TBRF IB positive sera in lanes 1 to 3 also demonstrated variable recognition of BipA and GlpQ proteins from different RFB species. 

## 4. Discussion

Because diagnosis by clinical symptoms alone is a challenging process, the serological detection of antibodies plays an important role in the diagnosis of LD, a borreliosis that is closely related to TBRF [25,27,28]. Pathogen-specific serum antibodies can be due to an ongoing infection or a recently resolved infection and this fact has to be taken into consideration in clinical interpretation. Different species of *Borrelia* employ a variety of mechanisms including antigenic variation to avoid and subvert the host immune response [30,31,53,54,55] as do other pathogens in blood, like malaria parasites [56], that can also cause chronic human infections. The many mechanisms that interfere with human immune responses in TBRF and LD can result in concentrations of antibodies below the threshold of detection, thus, confounding serological diagnosis. For example, 332 (5.6%) of a cohort of 5964 patients tested for LD were positive only by PCR and not by the WB serological test recommended by the CDC [47]. The delay between infection and the formation of detectable levels of antibodies is another factor that influences serological diagnosis. Our findings also suggest that the expected temporal pattern of switching from IgM to IgG antibody formation does not always occur in TBRF. This can further impact on the serological diagnosis of TBRF. 

### 4.1. Specificity of TBRF IBs

Immune rabbit antisera against common European and US LDB species that give positive reactions in LD IBs [34] only recognized RFB FlaB and none of the other RFB antigens in TBRF IBs, including fHbp where there is weak sequence homology between European LDB and *B. hermsii* proteins. Conversely, immune rabbit antisera to the two RFB species, *B. hermsii* and *B. coriaceae*, only showed a weak cross-reaction with the 41kDa LDB FlaB in a LD IB and did not react with other antigens used for scoring a positive reaction in LD IBs [34]. Together with our patient serum results, these findings suggest that apart from the known cross-reaction with the FlaB [21,34], there is no cross-recognition of other test antigens used in TBRF and LD IBs. The TBRF and LD IBs can, therefore, differentially detect human antibodies to RFB and LDB with appropriate scoring algorithms. 

False positive reactions in LD WB and IB assays with the CDC criteria for positivity have been observed in some cases of syphilis (caused by a related spirochete, *Treponema pallidum* subspecies *pallidum*)**, multiple sclerosis, and autoimmunity or allergic disease [34,57,58]. The scoring algorithm adopted here for TBRF IBs, however, yielded an estimated clinical diagnostic specificity of 99.5%. The only false positive reaction observed in the TBRF IB in the present study was with an autoimmune serum containing high concentrations of IgG where the presence of nonspecific antibodies can be expected. Sera from patients with many other tick-borne diseases, including LD, did not react positively in TBRF IBs. FlaB is a useful indicator for infection with *Borrelia* species because FlaB from other pertinent human pathogens such as *T. pallidium*, *T. denticola*, and *E. coli* show lower sequence homologies. FlaB was, therefore, used in our algorithm as a compulsory scoring antigen for increasing specificity for the detection of borreliosis, however, only BipA, GlpQ, and fHbp, but not FlaB, were used to discriminate between RFB and LDB reactivity in the present study. 

### 4.2. Sensitivity of TBRF IBs

Our findings show that sera of patients with active RFB infection demonstrated by a positive PCR test do not always contain antibodies detectable in TBRF IB assays. The estimated sensitivity of 70.5% for TBRF IBs may be low because of one or more of the confounding factors outlined above. The observation that clinical diagnostic parameters of the TBRF IBs are improved when second serum samples from the initially qPCR test-positive patients obtained after an interval of several weeks were tested is important. It suggests that repeating the TBRF IBs with subsequent sera is helpful in patients suspected to have TBRF but who may be negative by TBRF IBs or even blood smear examination and PCR tests. A limitation of the present study was that the detection of antibodies in TBRF IBs could not be related to clinical details of infection, illness or medical treatment. Such relationships merit future investigation. 

Detection of IgM and IgG antibodies separately is an established procedure in the serological diagnosis of LD that is expected to provide information on temporal characteristics of infection [3,29,34], however, our findings in TBRF patients show that IgM and IgG antibody formation against BipA, GlpQ, fHbp, and FlaB is variable, for example, only IgM antibodies may be detected against one antigen and only IgG antibodies against another in a particular serum, and that this pattern may change with a subsequent serum sample obtained several weeks later. Therefore, detection of either IgM or IgG antibodies against each of the antigens BipA, GlpQ, fHbp, and FlaB was chosen for scoring a positive reaction for TBRF. 

An additional complication for serological diagnosis of TBRF is that infections can be caused by different species of RFB. Patients may have acquired TBRF abroad, and thus be infected with RFB species that are not common in the country of their normal residence. Our findings with rabbit antisera show that there is variable cross-reactivity between BipA, GlpQ, and fHbp proteins from different RFB species. Therefore, inclusion of recombinant proteins from different RFB species in the TBRF IBs may allow for species and strain-specific differences in antigenicity and the detection of antibodies generated by infection with a broad range of RFB. Consequently, a reaction with one or more of the proteins within the target antigen groups BipA, GlpQ, fHbp, and FlaB was taken to indicate a positive reaction for that antigen group in scoring TBRF IBs. 

*Borrelia hermsii* and *B. coriaceae* are RFB species that, respectively, infect humans [13,14] and bovids [59], while *B. turcica* is a related *Borrelia* species that infects tortoises [60]. The recognition of all antigens used in scoring TBRF IBs by the rabbit antiserum to *B. hermsii*, and all but one of the antigens by the rabbit antisera to *B. coriaceae* and *B. turcica*, suggests that the test antigens are likely to be recognized to varying degrees by antibodies in patient sera generated against other, more closely related human-infecting RFB species. Further investigations of the other human-infecting RFB species are needed to unequivocally demonstrate such cross-reactivity within BipA, GlpQ, and fHbp, and a limitation to our studies has been the unavailability of relevant cultured RFB to raise antibodies in rabbits. 

IBs have many advantages over WBs. Examples include the following: (i) identifying infections with different RFB species because using a panel of WBs with cell lysates from all the common RFB species would be expensive and impractical for clinical use; (ii) WBs suffer from the disadvantage that irrelevant proteins can migrate close to test proteins and the levels of expression of test proteins may vary with culture conditions; and (iii) antigenic cross-reactions between numerous non-scoring proteins present in whole cell lysates that are shared between LDB, RFB, and related bacteria are eliminated from consideration in IBs. IBs, therefore, constitute a more standardized and inclusive method for detecting antibodies in TBRF. It could also be useful to evaluate TBRF IBs for diagnosing LBRF. 

Poor reactivity with RFB-specific rabbit antibodies and patient sera indicates that BmpA and OspC from RFB are not useful as test antigens in TBRF IBs. The continuing identification of new species of RFB in the USA and elsewhere, however, suggests that the TBRF IB format may need to evolve to address newly discovered RFB species. 

### 4.3. Identifying Infecting RFB Species from TBRF IB Findings

It has been suggested that because *B. turicatae* BipA does not cross-react with *B. hermsii* BipA, serum reactivity with *B. turicatae* BipA can be used to diagnose infection with *B. turicatae* [41]. However, the present findings suggest that identifying infecting species of RFB in TBRF IBs may not be possible using only one target antigen because individual patient sera, as well as the immune rabbit sera, showed variable recognition of BipA, GlpQ, and fHbp proteins from different RFB species. Further studies involving PCR-dependent species identification and the use of additional RFB-specific antigens for scoring IBs are needed to investigate the potential of TBRF IBs for identifying RFB species causing infections. 

### 4.4. Implications of the Findings for the Epidemiology of TBRF and LD

The IB assay findings in patients with LD symptoms show that the prevalence of TBRF in the USA may be underestimated due to misdiagnosis as LD, a conclusion compatible with findings utilizing TBRF WBs prepared from RFB whole cell lysates and PCR-based tests [25,37,45,61,62,63]. It reflects the difficulties physicians face in differentially diagnosing LD and TBRF using only clinical signs and symptoms and patient histories [25,27,28]. TBRF IBs can, therefore, meet the well acknowledged need for an antibody assay to help diagnose TBRF and differentiate it from LD [37]. 

TBRF IBs can also help advance knowledge about the epidemiology of TBRF. The ability of the TBRF IBs to detect antibodies in sera of patients in Australia and Ukraine shows potential worldwide applicability. There is evidence for LD and TBRF in Ukraine [64]. Indigenous transmission of LD and TBRF in Australia has been controversial [9,10,11], however, the present findings show that antibodies to both RFB and LDB are found in patients in Australia with symptoms of LD. While it is possible that this may be due to infections with RFB and LDB acquired in overseas countries, the findings show the need for more detailed investigations of the clinical aspects of infections and the possibility that both groups of *Borreliae* may be transmitted within Australia. The results also suggest that coinfections or co-exposure to LDB and RFB occurs and can be a global phenomenon in habitats where both responsible tick vectors are present. The implications of coinfections for patient treatment and management require further investigation, however, the use of TBRF and LD IBs in parallel advances the differential diagnosis of the two diseases in areas where both are endemic. Laboratory diagnosis by microscopy, PCR or culturing for *Borrelia* have drawbacks that have been outlined in the introduction. The use of a panel of different laboratory tests in addition to the IBs described here may be advantageous in resolving diagnosis in difficult cases of borreliosis.

### 4.5. TBRF and Malaria in Africa

Some TBRF species may produce intermittent high fevers, hepatomegaly, splenomegaly, and anemia which are symptoms that are also common in malaria [56]. Patients with TBRF due to *B. duttonii* and *B. crocidurae* in Africa have hence been frequently mistaken to have malaria and treated with antimalarial drugs [65]. The use of appropriate TBRF IBs can prevent such misdiagnosis in areas where malaria and TBRF are both endemic. 

## 5. Conclusions

The development of a line IB assay for the serological diagnosis of TBRF is described here for the first time. The TBRF IB may offer a one-step serological test that can be applied globally to support the diagnosis and epidemiological studies of TBRF. 

## 6. Patents

US Patent application 15/916717 titled “Species-Specific Antigen Sequences for Tick-Borne Relapsing Fever (TBRF) and Methods of Use”.

## Figures and Tables

**Table 1:** Reference human sera for determining the specificity of the tick-borne relapsing fever immunoblots (TBRF IBs).

Source	Characteristic	Total No. of Sera
CDC reference set (*n* = 50)	Endemic area control	10
	Fibromyalgia	5
	Mononucleosis	5
	Multiple sclerosis	5
	Non-endemic area control	10
	Periodontitis	5
	Rheumatoid arthritis	5
	Syphilis	5
NYB reference set (*n* = 21)	RPR +ve	8
	Epstein-Barr virus infection	4
	HIV-1 infection	4
	Cytomegalovirus infection	5
CAP and NYSHD reference set for autoimmunity and allergy (*n* = 42)	ANA +ve	2
	ANA -ve	2
	Elevated IgG	13
	Elevated IgE	4
	Normal IgE	2
	Anti-DNA antibody +ve	2
	RF +ve	9
	RF -ve	8
Columbia University (*n* = 12) Lyme disease [46]	Clinical Lyme disease and positive by the CDC two-tier test for antibodies to *Borrelia burgdorferi* ss	12
IGeneX (*n* = 87)	*Bartonella henselae* infection	7
	Human granulocytic anaplasmosis	16
	*Babesia microti* infection	14
	*Babesia duncani* infection	41
	Human monocytic ehrlichiosis	5
	Healthy controls	4

ANA: antinuclear antibodies; CAP: College of American Pathologists; CDC: Centers for Disease Control and Prevention; HIV: human immunodeficiency virus; NYB: New York Biologics, Southampton, NY, USA; NYSH: New York State Department of Health; RF: rheumatoid factor; RPR: rapid plasma reagin test for syphilis; ss: sensu stricto.

**Table 2:** TBRF IB results with sera from 51 patients positive by qPCR for RFB.

Serum Group	Number of Sera	Sera Scored Positive Only IgM IBs ^a^	Sera Scored Positive Only IgG IBs ^b^	Sera Scored Positive Individually in Both IgM & IgG IBs ^c^	Total Number of Sera Scored Positive in IgM and/or IgG IBs ^d^ (%)	Total Number of Sera Scored Positive for Either IgM or IgG Antibodies to Any of the Four Scoring Antigens (%) ^e^
**Comparison A**						
First serum sample from 16 patients who provided two samples	16	7	0	2	9 (56.3%)	9 (56.3%)
First serum sample from the other 35 patients who provided only one sample	35	9	9	5	23 (65.7%)	27 (77.1%)
Total	51	16	9	7	32 (62.7%)	36 (70.6%)
**Comparison B**						
Second serum sample from 16 patients who provided two samples	16	8	0	4	12 (75.0%)	12 (75.0%)
First serum sample from the other 35 patients who provided only one sample	35	9	9	5	23 (65.7%)	27 (77.1%)
Total	51	17	9	9	35 (68.6%)	39 (76.5%)

First serum samples from 51 patients were found to be qPCR positive for RFB. A second serum sample was received from 16 of the 51 patients after a period of 5 to 53 weeks after the first serum sample. ^a^ Number of sera positive only in IgM TBRF IB using the criterion of 2 of 3 antigens BipA (*Borrelia* immunogenic protein A), GlpQ (glycerophosphodiester diesterase), and fHbp (factor H binding protein) being recognized plus FlaB (p41 flagellin B). ^b^ Number of sera positive only in IgG TBRF IB using the criterion of 2 of 3 antigens BipA, GlpQ, and fHbp being recognized plus FlaB. ^c^ Number of sera positive in both IgM and IgG TBRF IB using the criterion of 2 of 3 antigens BipA, GlpQ, and fHbp being recognized plus FlaB in both IBs. ^d^ Sum of sera scored positive by applying the criteria in ^a–c^ for IgM and IgG TBRF IBs with values in parentheses showing the percentage positive of the total number of sera tested in the category. ^e^ Number of sera positive by applying the criterion of either IgM or IgG antibodies reacting with any of 2 of 3 antigens BipA, GlpQ, and fHbp plus additionally with FlaB (algorithm described in Section 3.2.1) with values in parentheses showing the percentage positive of the total number of sera tested in the category.

**Table 3:** Estimated clinical parameters for detecting antibodies in TBRF IBs.

Parameter	Estimate (95% CI) with Only First Sera	Estimate (95% CI) with Second Sera Where Available
Sensitivity	70.5% (56.0–82.1)	76.5% (62.2–86.8)
Specificity	99.5% (97.0–100)	99.5% (97.0–100)
PPV	97.3% (84.2–99.9)	97.5% (85.3–99.9)
NPV	93.4% (89.1–96.1)	94.6% (90.6–97.1)

PPV: positive predictive value; NPV: negative predictive value; CI: confidence interval.

**Table 4:** TBRF and Lyme disease (LD) line immunoblot findings in sera of patients with symptoms of LD.

Serum Group	Australia	Ukraine	USA
Total number of sera tested	100	121	1158
(i) Only TBRF IB positive	13	4	126
(ii) Only LD IB positive	21	20	100
(iii) Both LD and TBRF IB positive	3	4	76

## Supplementary Materials

The following are available online at https://www.mdpi.com/2227-9032/7/4/121/s1. Table S1: Pertinent sequence homologies for the scoring RFB proteins in TBRF IBs.

## List of Abbreviations

Bbss: *Borrelia burgdorferi sensu stricto*; BipA: *Borrelia* immunogenic protein A; CDC: US Centers for Disease Control and Prevention; EIA: enzyme-linked immunosorbent assay; fHbp: factor H binding protein; FlaB: p41 flagellin B; GlpQ: glycerophosphatediester diesterase; IB: immunoblot; LD: Lyme disease; LDB: Lyme disease *Borreliae*; LBRF: louse-borne relapsing fever; NCBI: National Centre for Biotechnology Information; NPV: negative predictive value; PCR: polymerase chain reaction; PPV: positive predictive value; qPCR: quantitative polymerase chain reaction; RF: relapsing fever; RFB: relapsing fever *Borreliae*; TBRF: tick-borne relapsing fever; WB: Western blot.
