# Supplementary material for: Line Immunoblot Assay for Tick-Borne Relapsing Fever and Findings in Patient Sera from Australia, Ukraine and the USA
Source: Healthcare (Basel). 2019 Oct 21;7(4):121. doi: 10.3390/healthcare7040121 (PMC6955669; doi:10.3390/healthcare7040121)
Supplement: Supplementary file 1 [file healthcare-07-00121-s001.pdf]

Supplementary Materials:

Table S1. Pertinent sequence homologies for the scoring RFB proteins in TBRF IBs.

Species	E-Value	%BLASTp Homology	GenBank Accession Number
Borrelia hermsii **FlaB (WP_012421925.1) protein homologues in pertinent bacteria**			
B. parkeri	0	94	WP_025375191.1
B. turicatae	0	94	WP_011772107.1
B. coriaceae	0	92	WP_025407827.1
B. turcica	0	90	WP_120103961.1
B. miyamotoi	0	92	WP_020954538.1
B. burgdorferi	0	92	WP_002556748.1
B. recurrentis	0	90	WP_012538694.1
B. lonestari	0	87	AAW 38937.1
Treponema pallidium	8.00E-71	39	CAA 45382.1
Treponema denticola	3.00E-70	39	WP_002668950.1
Escherichia coli	6.00E-40	32	WP_096282488.1
Borrelia hermsii **BipA (ADF49614.1) protein homologues in pertinent bacteria**			
B. turicatae	3.00E-98	41	AGN 32383.1
B. parkeri	3.00E-82	55	AHF 45615.1
B. duttoni	7.00E-64	47	WP_012539436.1
B. crocidurae	2.00E-64	47	WP_025401306.1
B. recurrentis	7.00E-61	46	WP_012539315.1
B. coriaceae	4.00E-53	32	AHH11081.1
B. miyamotoi	4.00E-24	40	WP_099591036.1
B. turcica	3.00E-20	34	WP_120104729.1
B. garinii	2.00E-07	27	WP_ 032986216.1
B. burgdorferi ss	4.00E-06	25	ACO 38445.1
E. coli taxid	No significant homologues identified		
Treponema taxid	No significant homologues identified		
Borrelia hermsii **GlpQ (ADD63790.1) protein homologues in pertinent bacteria**			
B. turicatae	0	89	AAG 24363.1
B. miyamotoi	0	89	WP_025443886.1
B. parkeri	0	89	AAG 24362.1
B. coriaceae	0	88	AAG 24364.1
B. turcica	0	86	WP_120104645.1
B. recurrentis	2.00E-175	89	AHM 02189.1
E.coli	2.00E-115	49	WP_001400696.1
Treponema pallidium	3.00E-83	39	WP_010881706.1
Treponema denticola	3.00E-14	30	WP_002671949.1
B. afzelli	1.1	35	ACJ 73522.1
B. burgdorferi ss	No significant homologues identified		

Species	E-Value	%BLASTp Homology	GenBank Accession Number
***Borrelia hermsii* fHbp (WP_025407000.1) protein homologues in pertinent bacteria**			
B. turicatae	6.00E-68	62	WP_020282240.1
B. parkeri	8.00E-66	62	CBJ 18717.1
B. miyamotoi	6.00E-52	52	WP_084821552.1
B. valaisiana	2.00E-45	56	WP_015899234.1
B. coriaceae	2.00E-44	52	WP_025408527.1
B. recurrentis	1.00E-36	42	CAV 05787.1
B. bavariensis	4.00E-31	40	WP_123772075.1
B. afzelli	4.00E-30	40	WP_011703930.1
B. burgdoferi ss	No significant homologues identified		
E. coli taxid	No significant homologues identified		
Treponema taxid	No significant homologues identified		
